# Considerations on Baseline Generation for Imaging AI Studies Illustrated on the CT-Based Prediction of Empyema and Outcome Assessment

**DOI:** 10.3390/jimaging8030050

**Published:** 2022-02-22

**Authors:** Raphael Sexauer, Bram Stieltjes, Jens Bremerich, Tugba Akinci D’Antonoli, Noemi Schmidt

**Affiliations:** 1 Department of Radiology and Nuclear Medicine, University Hospital Basel, 4031 Basel, Switzerland; bram.stieltjes@usb.ch (B.S.); jens.bremerich@usb.ch (J.B.); noemi.schmidt@usb.ch (N.S.); 2 Department of Informatics, Division of Research and Analytical Services, University Hospital Basel, 4031 Basel, Switzerland; tugba.akincidantonoli@usb.ch; 3 Department of Radiology, University Children’s Hospital Basel, 4056 Basel, Switzerland

**Keywords:** empyema, computed tomography, pleural findings, AI, outcome

## Abstract

For AI-based classification tasks in computed tomography (CT), a reference standard for evaluating the clinical diagnostic accuracy of individual classes is essential. To enable the implementation of an AI tool in clinical practice, the raw data should be drawn from clinical routine data using state-of-the-art scanners, evaluated in a blinded manner and verified with a reference test. Three hundred and thirty-five consecutive CTs, performed between 1 January 2016 and 1 January 2021 with reported pleural effusion and pathology reports from thoracocentesis or biopsy within 7 days of the CT were retrospectively included. Two radiologists (4 and 10 PGY) blindly assessed the chest CTs for pleural CT features. If needed, consensus was achieved using an experienced radiologist’s opinion (29 PGY). In addition, diagnoses were extracted from written radiological reports. We analyzed these findings for a possible correlation with the following patient outcomes: mortality and median hospital stay. For AI prediction, we used an approach consisting of nnU-Net segmentation, PyRadiomics features and a random forest model. Specificity and sensitivity for CT-based detection of empyema (*n* = 81 of *n* = 335 patients) were 90.94 (95%-CI: 86.55–94.05) and 72.84 (95%-CI: 61.63–81.85%) in all effusions, with moderate to almost perfect interrater agreement for all pleural findings associated with empyema (Cohen’s kappa = 0.41–0.82). Highest accuracies were found for pleural enhancement or thickening with 87.02% and 81.49%, respectively. For empyema prediction, AI achieved a specificity and sensitivity of 74.41% (95% CI: 68.50–79.57) and 77.78% (95% CI: 66.91–85.96), respectively. Empyema was associated with a longer hospital stay (median = 20 versus 14 days), and findings consistent with pleural carcinomatosis impacted mortality.

## 1. Introduction

Artificial intelligence offers multiple new possibilities for quantitative image analysis in radiology. AI-aided anatomical segmentation, such as lung segmentation for quantification of lung infiltrates [1], is already established in clinical routines. AI also holds great promise in classifying different pathologies [2]. However, there are major challenges regarding the classification of diseases: In order to train and evaluate an algorithm, high diagnostic accuracy is required for disease classification, but CT-based radiological diagnosis often provides only moderate diagnostic accuracy, depending on the clinical question. Conversely, AI-based quantification or classification is of particular interest for those diagnoses with only moderate radiological diagnostic accuracy. For the training, validation and testing of an AI model, high demands should be made of the reference standard (“ground truth”). Since the primary goal of the development of AI tools should be the application of these tools in routine clinical practice, a classifier should be developed on a heterogeneous dataset that is as independent from the “inclusion” and “exclusion criteria” as possible. This need for generalizability is often in contrast with published data. A large proportion of published diagnostic accuracy and outcome studies [3] shows limiting exclusions of diagnostically challenging cases.

Empyemas are pleural effusions with pus in the pleural space and are most commonly secondary to pneumonia [4]. While empyema-related hospitalizations increase [5], empyemas are additionally associated with worse outcomes, such as prolonged admission, more complications [6] and therefore more invasive management [7] compared to parapneumonic effusions. Distinguishing empyema from other forms of pleural effusion is radiologically challenging; an AI-based classification of effusions could help in the imaging reading process. CT is an integral part of routine clinical diagnostics for the timely diagnosis of empyema; however, there is a large heterogeneity of published diagnostic accuracy measures [8]. These differences might be explained by small sample sizes, differences in reference standards, CT-acquisition, and publication date. Currently, there is no diagnostic accuracy study for the diagnosis of empyema (instead of CT features), nor is there an investigation of outcome measures based on radiological reporting.

The main objective of this study is to generate a dataset for an AI-classifier for detecting empyema in pleural effusions based on routinely performed CTs with pathological confirmation in combination with outcome predictors. The first aim is to (1) determine the diagnostic accuracy of “empyema” and the reported pleural CT features in routinely acquired radiological reports. The second aim is to ascertain the diagnostic accuracy of “pleural CT features” in a blinded manner (2). The third aim is to define a consensus based on routine radiological findings and the blinded interpretation as the reference standard and to evaluate this consensus based on sensitivity and specificity (3). Fourth, we aim to assess pleural features for their prediction of hospital stay time and mortality (4) and finally a prototype for automated empyema prediction is to be developed.

## 2. Materials and Methods

This study was approved by the local ethics committee (Project ID: 2021-00946) and is registered on the German Clinical Trials Register (DRKS00025201). No protocol deviation occurred.

### 2.1. Participants

#### 2.1.1. Eligibility Criteria

Eligible patients were retrospectively identified based on the presence of pleural effusion in the radiological report between 1st January 2016 and 1st January 2021. All routine chest CTs were included regardless of contrast phase, with pathological reports within 7 days. Patients without pathologic reports and follow-up examinations were excluded. To avoid an inappropriate exclusion, patients who had already received a chest tube for volume decompression prior to CT were not excluded. Additionally, hospital stay time, final diagnosis, and presence of death until April 2021 were extracted from patient records.

#### 2.1.2. Intended Sample Size

We calculated the sample size with an estimated empyema prevalence of 10% in parapneumonic effusions (power = 0.8; *p* < 0.5; H0: 0.7; H1: 0.9), with a minimum total number of 310 patients for sensitivity (min. empyema: 31) and 34 for specificity (min. empyema: 3). A total of 335 patients with pathological correlation could be identified in the hospital database for the study period between January 2016 and January 2021, and we decided to include the entire consecutive cohort in the study.

### 2.2. CT and Acquisition

Scans were acquired using three different CT scanners: Somatom Definition Flash (*n* = 95, 2 × 128-slice system), Somatom Definition AS+ (*n* = 182, 128-slice system), and Somatom Definition Edge (*n* = 58, 128-slice system; all scanners: Siemens Healthineers, Erlangen, Germany). The peak kilovoltage was 120 kVp and an automatic tube current modulation was performed. A contrast agent was administered in 208 of the 335 CT studies, with routine flow rates of between 2 and 4 mL/s (84 biphasic). Soft tissue kernels (30f), with 1 mm acquisition and 5 mm reconstructions in the coronal, axial, and sagittal planes, as well as 0.7–1 mm lung kernels (70f) were used for image interpretation on EIZO RX350 (EIZO, Ishikawa, Japan) diagnostic monitors.

### 2.3. Pleural CT Features

**Figure 1:**
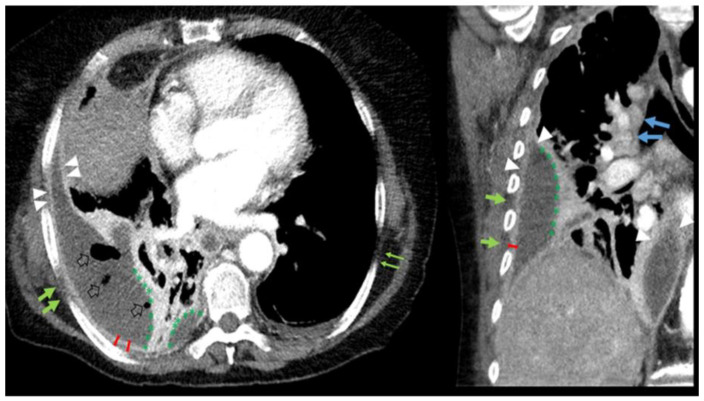
Axial (**right**) and coronal (**left**) reconstruction of a 73-year-old patient with empyema on the right side. The arrowheads show increased pleural enhancement of the parietal (costal and mediastinal) and visceral (lung) pleura, consistent with a “split pleura sign” associated with pleural thickening (red dash). Pleural fat stranding (bold green arrows, compared to the normal contralateral side, thin green arrows) and microbubbles (empty arrows) are also present. Pleural empyema on the right side is loculated (green *) in contrast to the simple pleural effusion on the contralateral side. There is reactive hilar and mediastinal lymphadenopathy (blue arrows).

The empyema-associated pleural CT features described in the literature are pleural thickening, pleural enhancement, microbubbles, extrapleural fat stranding, and loculation. Figure 1 shows an example of the typical features of an empyema.

#### 2.3.1. Radiological Report-Based CT Feature Extraction

Text-based, anatomically structured radiology reports, blinded by definition to the reference standard, were prospectively generated in consensus by a radiology resident and a board-certified specialist. The radiological diagnosis was routinely made based on image findings and knowledge of the clinical information. R.S. (4th post-graduate year, PGY; for details see Appendix A) extracted pleural CT features and test results for pleural empyema.

#### 2.3.2. Prespecified CT Based CT Feature Extraction

All CTs were interpreted independently by R.S. (4 PGY) and N.S. (10 PGY) concerning the following pleural CT features. The interpretation was blinded to radiological reports, clinical information, and pathological diagnosis.

Based on the literature, the aforementioned pleural CT features were divided into the following groups:

Pleural thickening was defined as a visible pleural line and classified based on location (circumferential, lung-, rib-, mediastinal involvement) and morphology (smooth, nodular (>2 mm, round), or pleural mass (>3 cm)).

Visible pleural enhancement was also scored as pleural thickening. Thus, the descriptors for location and morphology also apply to any pleural enhancement present. Visible pleural thickening without visible enhancement was not considered enhancement. Pleural enhancement was divided into split pleura sign (visible enhancement of both visceral and parietal pleura) and hemisplit pleura sign (visible enhancement of either visceral or parietal pleura).

Microbubbles were defined as gas surrounded by pleural fluid.

The extrapleural fat stranding was defined as having higher HU values compared to the contralateral side and the subcutaneous fat tissue.

We defined loculation as obtuse angles with the lung (>90°; <180°). Interlobular fluid was defined as fluid within the interlobular fissure.

According to Tsujimoto et al., a cutoff of 3 cm (the maximum measured distance on an axial slice) was used for the amount of pleural effusion [9]. Rib destruction was defined as osteolysis adjacent to pleural effusion. Mediastinal/hilar lymphadenopathy was defined by a short axis > 1 cm.

After evaluation of the interrater agreement, the non-consensus was resolved by J.B. (29 PGY).

### 2.4. Reference Standard

As the reference standard, we used the final pathology report within 7 days of the CT. The reports describing macroscopic pus or fibropurulent changes were rated as positive for empyema according to literature [10,11,12]. Additionally, macroscopic or microscopic pleural tumor manifestations were defined as pleural carcinomatosis. Clinical information and index test results were available via the hospital information system.

### 2.5. Possible Applications

We used an nnU-net architecture [13] for 3D pleural effusion segmentation of the dataset. In order to evaluate the extent to which radiomic features could be suitable for predicting an empyema, we used the Python software (version 3.7) and the PyRadiomics package for feature extraction [14]. In the preselection phase, we selected 50 features with the highest importance among all the extracted radiomic features. Then a random forest model with bootstrap sampling and 100 decision trees was trained based on a leave-one-out cross-validation, balanced 1:1 regarding pathologically confirmed empyema (*n* = 81) and randomly selected negatives. Finally, the model was applied to all (*n* = 335) cases to evaluate prediction performance.

### 2.6. Analysis

To test for normal distribution, we used the Kolmogorov–Smirnov test (e.g., patients’ age). We used a *t*-test to compare the differences in patients’ ages between the positive and negative collectives. Interrater variability was assessed with Cohen’s kappa and waived by consensus with a third rater (J.B.). Test results were organized into 2 × 2 contingency tables, displaying true positives, true negatives, false positives, and false negatives. Pearson’s-Chi (with Cramer’s V), sensitivity, specificity, negative predictive value (NPV), positive predictive value (PPV), accuracy, diagnostic odds ratio (DOR), area under the curve (AUC) as well as 95% CI intervals, were calculated for each pleural CT feature. We used Mann–Whitney-U for the analysis of hospital stay time and performed Kaplan–Meier analysis for survival time. All statistical analyses were performed with R 4.0.5 (R Core Team, Vienna, Austria).

## 3. Results

### 3.1. Study Population

**Figure 2:**
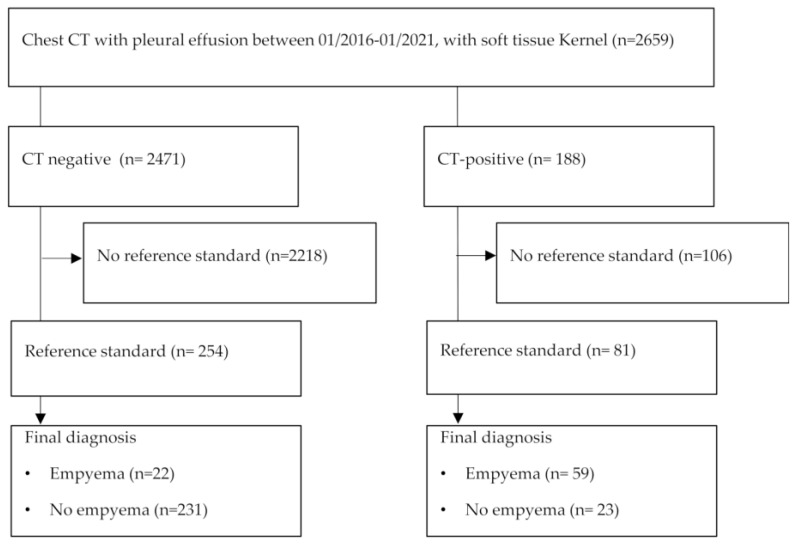
Study flow chart according to STARD [16].

A total of 2659 eligible patients were identified with pleural effusions between 01/2016 and 01/2021. Of these, 335 patients had pathology workup within 7 days of computed tomography regardless of effusion cause or underlying disease (see Figure 2) and their results are available for download [15].

**Figure 3:**
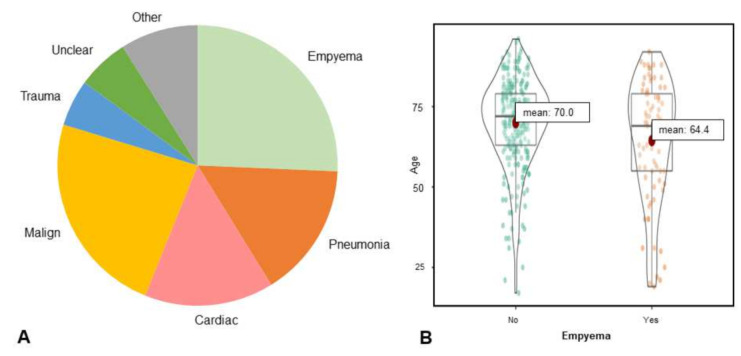
(**A**). The pie chart summarizes the distribution of the different pleural effusion causes (**B**). Shows the age distribution in the dataset.

Of the 335 patients included, 125 were female (37.3%). The mean age was 68.6 years (95-CI: 67.0–70.3, Median: 71, range: 18–96). The primary etiologies (see Figure 3) of pleural effusion were empyemas (*n* = 81), pleural malignancy (*n* = 60, with malignant cells) and others (n = 194). Other leading causes of pleural effusion were pneumonia (*n* = 52), acute or chronic heart failure with pulmonary congestion (*n* = 50), and trauma (*n* = 18). The most common malignancy with associated pleural effusion was lung cancer. Pleural carcinomatosis was confirmed by pathology in 34 patients. In 20 cases, the etiology of pleural effusion remained unclear.

Pathology diagnoses were based either on intra-operative samples (*n* = 61), biopsies (*n* = 42), or fine needle aspiration or thoracentesis (*n* = 231). A total of 82 patients with empyema were identified. In 14 empyema cases, malignant cells were additionally detected in the pathological specimen with known underlying malignancy.

The patients with empyema were slightly younger (mean age 64.4 versus 70.0, t = 2.87, *p* = 0.004). In the subset of patients with empyema, 33.8% were women versus 38% in the subset without empyema. Contrast medium was administered in 79% of the cases.

### 3.2. Estimates of Diagnostic Accuracy

#### 3.2.1. Diagnostic Accuracy Based on Radiology Report

Sensitivity and specificity of empyema diagnosis were 72.84% (95% CI: 61.63–81.85) and 90.94 (95% CI: 86.55–94.05). After contrast administration, sensitivity and specificity were higher (75.00% (95% CI: 62.35–84.62); 86.81% (95% CI 79.91–91.67), respectively). AUC, NPV and PPV were 0.84 (95% CI: 0.79–0.90), 91.30% (95% CI: 86.96–94.35%) and 71.95% (95% CI: 60.77–81.04%), respectively.

Diagnostic accuracy was higher if only benign effusions were considered (sensitivity: 73.75% (95% CI: 62.52–82.67%); 91.86 (95% CI: 92.96–97.83%). The radiological sensitivity and specificity for pleural carcinomatosis were 70.59 (95% CI: 52.33–84.29) and 96.01 (95% CI: 92.96–97.83%), respectively.

#### 3.2.2. Interrater Agreement

Regarding pleural features with known association with pleural empyema, we found a substantial agreement (mean kappa = 0.66) between both readers (pleural thickening: circumferential = 0.66, lung = 0.41, rib = 0.73, mediastinal = 0.71, smooth = 0.65; pleural enhancement: hemi- split = 0.77, split pleura sign = 0.79, microbubbles = 0.82, extrapleural fat stranding = 0.48, loculation = 0.62), whereas thickening of the visceral pleura and fat stranding were most difficult to assess. Table 1 shows Cohen’s kappa for individual pleural features.

#### 3.2.3. Diagnostic Accuracy to Differentiate Pleural Empyema from Other Effusions

The 95%-CI diagnostic odds ratio (DOR) from all the aforementioned pleural CT features did not include 1, except pleural nodularity, pleural mass, amount, and rib destruction which are summarized in Table 2. Pleural thickening showed an overall accuracy of 81.49%, a sensitivity of 70.38% (95% CI: 59.04–79.74%), and a specificity of 85.04% (95% CI: 79.92–89.07%). Nodular thickening or a pleural mass were atypical of empyema with an NPV of 74.68% (95% CI: 69.44–79.31%) and 75.15% (95% CI: 70.00–79.70%), respectively. When only CTs after contrast administration are considered, pleural enhancement showed the highest accuracy, with 87.02. The split pleura sign was more specific for diagnosing empyema (93.06%, 95% CI: 87.26–96.43, sensitivity: 59.38%, 95% CI: 46.38–71.24%). Circumferential pleural thickening/pleural enhancement showed an accuracy of 83.58%, which is comparable to the split pleura sign (82.69%). While microbubbles and extrapleural fat stranding had high specificity (91.34%, 95% CI: 87.01–94.37% and 90.94, 95% CI: 86.55–94.05%), loculation showed a higher sensitivity with 80.24% (95% CI: 69.61–87.95%). 28.06% of patients without empyema, 43.59% of patients with pneumonia, and 51.52% of patients with empyema showed accompanying hilar or mediastinal lymphadenopathy. Effusion volume was not specific for empyema (19.00%, 95% CI: 14.39–24.37, sensitivity: 82.72, 95% CI: 72.36–89.90). Diagnostic accuracy measures are summarized in Table 2.

### 3.3. Outcome Measures

**Figure 4:**
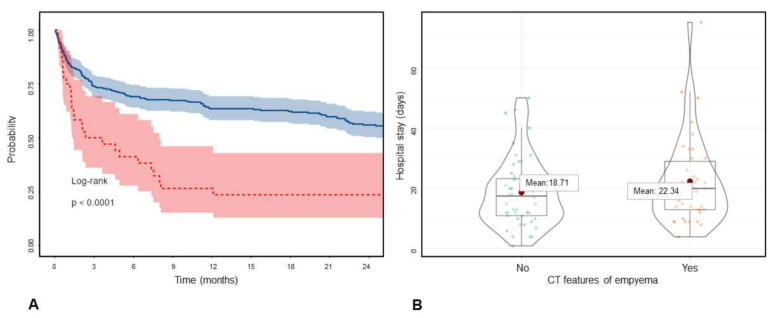
(**A**). Survival of the patients with (red) and without (blue) CT features of pleural carcinomatosis based on Kaplan-Meier survival analysis. (**B**). Hospitalization duration in pneumonia patients with and without CT features of empyema.

Among all 335 patients with pleural effusion, 51.04% died (*n* = 171). Pleural effusion in general had a worse prognosis, with a mean survival of 1103.56 days (95% CI: 890.27–1316.84) in cases without underlying malignancy and 1005.29 days (95% CI: 900.53–1110.05) in cases with known malignancy (χ2: 0.142, *p* = 0.706). Radiological intrathoracic tumor manifestation was associated with a poorer prognosis, with a mean survival of 573.83 days (95% CI: 402.21–745.45) versus 1132.03 days (95% CI: 1023.62–1240.45; median 131 versus 1318 days; χ2: 26.95; *p* = 0.000). Patients with radiological signs of pleura carcinomatosis (*n* = 36) had a shorter mean survival time (414.45 days: 95% CI: 212.81–616.08) versus 1061.91 days (95% CI: 961.38–1162.43, χ2: 11.535; *p* = 0.001, see Figure 4A).

Patients with pleural nodularity or mass had shorter mean survival, with 445 (95% CI: 147–734) and 432 (95% CI: 85–779) days compared to 1026 (95% CI: 928–1124) and 1018 (95% CI: 921–1115) days, respectively. Empyema did not show a higher mortality rate but was associated with increased length of hospital stay (20 versus 14 days median, *p* = 0.035), similar to pleural enhancement (*p* = 0.124). Figure 4B also shows this trend in empyema versus parapneumonic effusions. Outcome measures are summarized in Table 3.

### 3.4. Possible Applications

**Figure 5:**
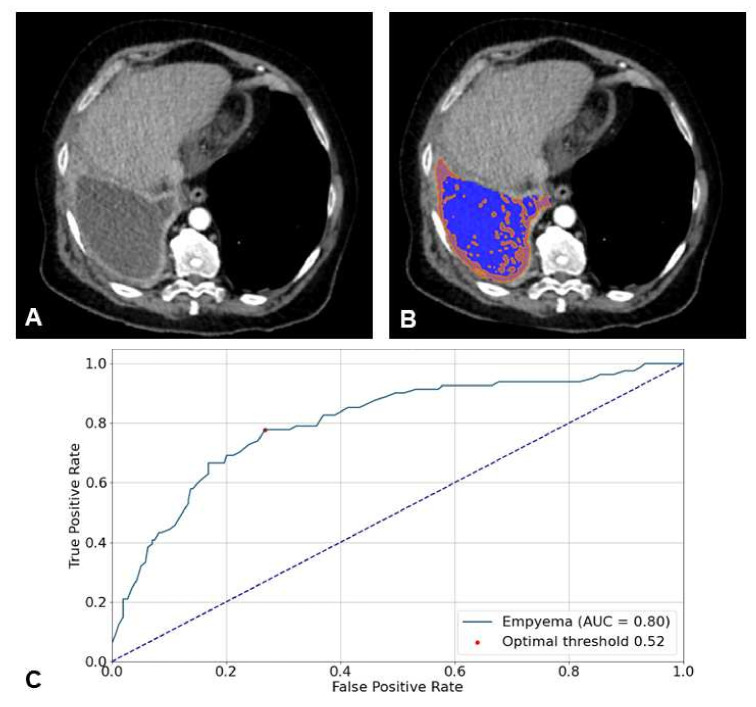
(**A**). Biphasic axial CT with pathologically proven empyema on the right side with pleural thickening and enhancement. (**B**). nnU-net based 3D segmentation (blue), within the mask density values > 15 HU are colored orange. (**C**). ROC analysis of the random forest model based on radiomics features to predict empyema. The optimal threshold is based on Youden’s index.

In addition to the CT datasets, the nnU-net based segmentation masks were published as well and are freely available [15]. Figure 5B shows a corresponding example, with higher density values depicted within the segmentation mask. It shows that density-based classification approaches could possibly be useful. The random forest model based on radiomics features performed with a sensitivity of 77.78% (95% CI: 66.91–85.96) and specificity of 74.41% (95% CI: 68.50–79.57) for the prediction of pleural empyema.

## 4. Discussion

The sensitivity and specificity of CT to diagnose an empyema in clinical practice are 72.84% and 90.94%, respectively. We found moderate to almost perfect interrater-agreement with a sensitivity of 70.37% for pleural thickening, 78.13% for pleural enhancement, 46.91% for fat stranding, and 80.24% for loculation, and corresponding specificities of 85.04%, 90.97%, 90.94% and 78.74%, respectively. The automated detection of pleural empyema achieved an AUC of 0.80.

With a total of 335 consecutive patients and 81 empyemas, this is the largest study regarding the total study population (*n* = 24 [17]–215 [18]) and patients with empyemas (9 [19]–58 [20]) publicly available containing all CT datasets, pleural 3D segmentation, radiological features, pathological reference standards, outcome information, and the random-forest-based radiomics classification model [15].

In comparison to previous studies [8], we found higher sensitivity for pleural thickening [21,22,23], pleural enhancement [10,18,20] and loculation [20,22] with comparable specificity. While most diagnostic accuracy studies used GE 8800 [19,20,24] or GE 9800 [17,19,23,24] scanners, this difference might be explained by newer CT scanner generations with higher resolution and shorter scanning times in our study (128-slice CT scanners).

Since the nomenclature is heterogeneous, we have attempted to use clear definitions for pleural findings based on published studies. While Jimenez et al. and Leung et al. described the anatomic location (e.g., visceral, parietal) of pleural thickening, Tsujimoto et al. [9] used the term “split pleura” sign for visceral and parietal pleural thickening regardless of contrast media, whereas Porcel et al. [18] retained the “split pleura” as a threshold for pleural enhancement. As we understand every visible pleural enhancement as visible pleural thickening, we reserved “enhancement”, “split- and hemisplit pleura sign” for contrast-enhanced CTs and added a more detailed anatomic description for pleural thickening. Our literature-based definitions might lead to a more standardized reporting nomenclature.

Additionally, this is the first study to evaluate the imaging-based diagnosis of empyema based on prospective gathered reports. We found a high negative predictive value (NPV) of 91.30%, which is comparable to the NPV of CT based diagnoses of COVID-19 pneumonia [25].

Whereas pleural effusions are known as negative outcome predictors in various disease entities, which holds true as well for the ongoing COVID pandemic [26], they show high one-year mortality rates in both non-malignant (25%–57% [27]) and malignant diseases (e.g., 77% [28]). This is consistent with our results. As pleural carcinomatosis has a poor prognosis [29], correlation with pleural findings associated with radiological manifestation is not surprising. With the improvements in patient management and adequate treatment of acute pleural diseases like empyema, the mortality rates have been reduced. However, pleural diseases are still leading to longer hospitalizations, which might be improved by early detection.

With this first radiomics-based study, we have shown that empyema is predictable with high accuracy and that the translation of known CT features based on 3D segmentation might be reasonable for AI algorithms. Comparable to other chest pathologies [30], the tools for risk calculation and outcome prediction are promising.

A limitation of the study is that eligible patients were retrospectively selected on scanners of one vendor at a single institution. A second limitation is that the reference standard was only applied if clinically indicated, hence the empyema prevalence might be higher than expected. Third, in addition to avoiding inappropriate exclusion, patients with chest tubes in situ were also included, which increased the prevalence of iatrogenic microbubbles. However, in contrast to a general tendency to exclude these patients, this better reflects the routine clinical setting. According to the current guidelines [31], only CT was used as a reference test in the current study; nevertheless, it might be worthwhile to investigate the diagnostic accuracy of other modalities such as FDG-PET in the future, which has already proved to be useful for other pleural diseases [32].

With the development of AI-based algorithms for disease detection and classification, outcome evaluation on diagnostic images might become increasingly relevant. We showed that known radiological descriptors vary in their potential for prognostication and can be used as a benchmark for automated tools.

## 5. Conclusions

This study serves as an update of previous diagnostic accuracy studies in terms of developments in biomedical engineering, and the results can contribute to more structured reporting. With an AUC of 0.84, the radiological diagnosis of empyema can help to identify patients with longer hospital stays. We hope that the openly available, anonymous CT data, the consensus-based CT features and pathological and outcome data [26] can be used as a baseline for further AI research.

## Figures and Tables

**Table 1:** Interrater Agreement.

	Kappa *
pleural thickening	
Overall	0.68
circumferential	0.66
Lung	0.41
Rib	0.73
Mediastinal	0.71
smooth	0.65
nodular	0.61
pleural mass	0.63
Enhancement *	
split pleura sign *	0.79
overall (incl. hemi split pleura sign) *	0.77
gas	0.75
microbubbles	0.82
pneumothorax	0.97
extrapleural fat stranding	0.48
loculation	0.62
amount	0.80
other findings	
rib destruction	0.87
blood	0.38
interlobar fluid	0.47
mediastinal lymphadenopathy	0.52

All *p*-values are ≤0.001. * 208 studies with contrast media including 64 empyemas (17 studies with empyema where without contrast media).

**Table 2:** Diagnostic accuracy of CT features.

	Chi^2^	FP	TN	TP	FN	Sensitivity (95% CI)	Specificity (95% CI)	DOR (95% CI)
pleural thickening								
overall	92.81 *	38	216	57	24	70.37 (59.04–79.74)	85.04 (79.92–89.07)	60.00 (39.68–90.73)
circumferential	84.69 *	4	250	30	51	37.03 (26.78–48.54)	98.42 (95.75–99.49)	52.08 (39.41–68.81)
lung	96.13 *	10	244	39	42	48.15 (37.02–59.46)	96.06 (92.66–98.00)	54.2 (39.62–74.14)
rib	103.69 *	10	244	41	40	50.62 (39.36–61.81)	96.06 (92.66–97.99)	57.08 (41.55–78.42)
mediastinal	77.03 *	7	247	31	50	38.27 (27.89–49.78)	97.24 (94.16–98.79)	48.46 (36.1–65.05)
smooth	120.54 *	21	233	54	27	66.67 (55.22–76.51)	91.73 (87.46–94.69)	69.33 (47.23–101.79)
nodular	3.93 *	18	236	1	80	1.23 (0.65–7.64)	92.91 (88.84–95.63)	0.21 (0.03–14.14)
pleural mass	2	12	242	1	80	1.23 (0.06–7.64)	95.28 (91.68–97.42)	0.31 (0.05–20.55)
enhancement **								
split pleura sign **	68.61 *	10	134	38	26	59.38 (46.38–71.24)	93.06 (87.26–96.43)	48.72 (33.3–71.28)
hemi split pleura sign **	112.65 *	13	131	50	14	78.13 (65.71–87.11)	90.97 (84.75–94.91)	82.2 (49.19–137.38)
gas	39.14 *	52	202	46	35	56.79 (45.33–67.60)	79.53 (73.93–84.21)	31.78 (21.93–46.08)
microbubbles	87.93 *	22	232	46	35	56.79 (45.33–67.60)	91.34 (87.01–94.37)	51.61 (36.37–73.22)
pneumothorax	16.71 *	47	207	33	48	40.74 (30.13–52.24)	81.49 (76.05–85.96)	21.91 (15.21–31.57)
extrapleural fat stranding	59.1 *	23	231	38	43	46.91 (35.85–58.27)	90.94 (86.55–94.05)	39.7 (28.34–55.59)
loculation	39.14 *	54	200	65	16	80.24 (69.61–87.95)	78.74 (73.09–83.50)	73.74 (44.76–121.47)
amount	0.106	206	48	67	14	82.72 (72.36–89.90)	19 (14.39–24.37)	10.87 (0.66–18.02)
other findings								
rib destruction	0.86	8	246	1	80	1.23 (0.06–7.64)	96.85 (93.66–98.53)	0.45 (0.07–29.02)
interlobar fluid	5.59 *	128	126	53	28	65.43 (53.96–75.43)	49.61 (43.32–55.91)	16.11 (10.75–24.13)
mediastinal lymphadenopathy	5.485 *	77	177	36	45	44.44 (33.55–55.88)	69.69 (63.57–75.19)	15.72 (10.8–22.87)
diagnosis								
empyema	135.163 *	23	231	59	22	72.84 (61.63–81.85)	90.94 (86.55–94.05)	82.74 (54.28–126.13)
pleura carcinomatosis	141 *	12	289	24	10	70.59 (52.33–84.29)	96.01 (92.96–97.83)	19.93 (10.39–38.25)

* *p* ≤ 0.05. ** 208 studies with contrast media including 64 empyemas (17 studies with empyema where without contrast media). FP: False positives. TN: True negatives. TP: True positives. FN: False negatives.

**Table 3:** Radiology and Outcome.

CT Features	Median Hospital Stay Time in All Patients				Survival Time (Kaplan-Meier-Analysis)			
pleural thickening	with (d)	without (d)	U	*p*	mean with in days	mean without (d)	χ2	*p*
overall	20	14	10514	0.319	1094 (846–1069)	957 (846–1069)	1.774	0.183
circumferential	23	15	4220	0.105	1191 (921–1463)	968 (867–1069)	2.485	0.115
lung	22	14	6221	0.236	1238 (1012–1464)	945 (842–1048)	6.141	0.013
rib	22	15	6094	0.083	1220 (982–1459)	955 (852–1059)	3.369	0.066
mediastinal	21	15	5006	0.283	1110 (844–1376)	976 (874–1077)	1.371	0.242
smooth	20	15	9117	0.447	1242 (1045–1441)	925 (818–1033)	7.27	0.007
nodular	20	15	2721	0.52	445 (147–734)	1026 (928–1124)	4.131	0.042
pleural mass	23	15	1828	0.459	432 (85–779)	1018 (921–1115)	3.6	0.057
enhancement *								
split pleura sign *	20	15	3097	0.048	1214 (991–1438)	1044 (906–1182)	1.88	0.17
overall (incl. hemi split pleura sign) *	20	14	3558	0.014	1193 (995–1391)	1032 (887–1176)	2.362	0.124
gas	19	14	10630	0.269	1009 (838–1181)	990 (875–1104)	0.055	0.815
microbubbles	20	14	7976	0.144	1106 (903–1308)	962 (855–1069)	1.017	0.313
pneumothorax	18	15	9931	0.801	909 (721–1098)	1025 (914–1135)	1.753	0.186
extrapleural fat stranding	21	14	7431	0.203	1008 (902–1093)	992 (886–1097)	0.002	0.969
loculation	19	13	12054	0.419	1141 (982–1300)	911 (793–1029)	4.951	0.026
amount	16	14	7072	0.052	978 (872–1084)	1076 (857.74–1295)	0.555	0.456
other findings								
rib destruction	10	15	1227	0.416	264 (6–522)	1013 (916–1110)	2.645	0.104
blood	15	15	142	0.34	642 (225–1059)	1005 (908–1103)	0.722	0.396
interlobar fluid	18	14	12162	0.064	988 (902–1093)	1009 (869–1150)	0.018	0.894
mediastinal lymphadenopathy	17	15	11960	0.572	894 (732–1056)	1040 (923–1156)	1.512	0.219
diagnosis								
empyema	20	14	8695	0.035	1257 (1074–1440)	911 (801–1021)	7.617	0.006
pleura carcinomatosis	17	15	4631	0.19	414 (212–616)	1062 (961–1162)	11.535	0.001

* 208 studies with contrast media including 64 empyemas (17 studies with empyema where without contrast media).

## Data Availability

The data presented in this study are openly available in zenodo.org at https://doi.org/10.5281/zenodo.5793365, reference number: 5793366.

## Appendix A

Based on the anatomically structured radiology reports, a description of the pleura was assured. However, neither the individual pleural CT features nor their thresholds were prespecified, and a uniform definition of pleural CT features was not available at the time of routine reporting. The radiological diagnosis was routinely made based on the image findings and knowledge of the clinical information.

Described pleural CT features and test results (empyema) based on differential diagnosis were extracted by R.S. and assigned to the subsumed CT features. A linguistic weighting of the diagnosis of at least 50% as differential diagnosis (“is likely”; “compatible with”; “diagnosis A versus diagnosis B”) was assessed as a positive test (both for pleural CT features and the diagnosis of empyema). A weighting of less than 50% (“unlikely”/“no evidence of”) was rated as a negative test. Cases with no description were rated as negative (e.g., no split pleural sign if no description of the pleural enhancement was available). Routine assessment of peripleural fat tissue was not done in the radiological reports and therefore could not be extracted.
